# Prevalence and associated factors of active trachoma among childeren aged 1–9 years in rural communities of Gonji Kolella district, West Gojjam zone, North West Ethiopia

**DOI:** 10.1186/s13104-015-1529-6

**Published:** 2015-11-04

**Authors:** Adane Nigusie, Resom Berhe, Molla Gedefaw

**Affiliations:** Department of Health Education and Behavioral Sciences, Institute of Public Health, College of Medicine and Health Science, University of Gondar, P.O. Box 196, Gondar, Ethiopia; GAMBY College of Medical Sciences, Bahir Dar, Ethiopia

**Keywords:** Active trachoma, Children, Gonji kollela, Prevalence, Risk factor

## Abstract

**Background:**

Trachoma is the leading infectious cause of blindness worldwide. Though trachoma can be treated with antibiotic it is still endemic in most part of Ethiopia.

**Methods:**

A community based cross-sectional study was conducted among 618 children 1–9 years of age from December 2013 to June 2014. A multistage systematic sampling technique was applied. Data were collected using pretested and structured questionnaire and also observation by using binocular loupe to differentiate active trachoma cases. The World Health Organization’s simplified classification scheme for assessing trachoma in community based surveys was used for the purpose. Bivariate and multivariate logistic regression model was fitted to identify factors associated with trachoma among children aged 1–9 years. An adjusted odds ratio with 95 % confidence interval was computed to determine the level of significance.

**Results:**

The overall prevalence of active trachoma among children aged 1–9 years were 23.1 % (Trachomatous inflammation—Follicular, in 22.5 % (95 % CI: 22.3–22.69 %); Trachomatous inflammation—Intense, in 0.6 % (95 % CI: 0.4–0.79 %). Family size (>5) (AOR = 14.32, 95 % CI = 6.108–33.601), number of children under 10 years of age within household (AOR = 25.53, 95 % CI = 9.774–66.686), latrine utilizations (AOR = 10.274, 95 % CI = 4.274–24.968), route of waste disposal (AOR = 3.717, 95 % CI = 1.538 to −8.981), household literacy (AOR = 2.892, 95 % CI = 1.447–5.780), cattle housing practice (AOR = 4.75, 95 % CI = 1.815–12.431), time to collect water (AOR = 25.530, 95 % CI = 8.995–72.461), frequency of face washing practice (AOR = 6.384, 95 % CI = 2.860–14.251) and source of water (AOR = 2.353, 95 % CI = 1.134–4.882) were found to be associated with the presence of active trachoma in this study population.

**Conclusion:**

The prevalence of active trachoma among rural communities of children aged 1–9 years was found to be high in reference to WHO recommended thresholds to initiate trachoma control recommendation (>10 % prevalence), which indicates that active trachoma is still a major public health concern in the study area. Therefore, it is recommended that coordinated work on implementing the WHO endorsed SAFE strategy in particular and enhancing the overall living conditions of the community is crucial.

## Background

Trachoma is one of the oldest infectious diseases known to mankind. It is caused by the bacterium *Chlamydia trachomatis* a microorganism which spreads through contact with eye discharge from the infected person (on towels, handkerchiefs, fingers, etc.) and through transmission by eye-seeking flies. After years of repeated infection, the inside of the eyelid may be scarred so severely that the eyelid turns inward and the lashes rub on the eyeball, scarring the cornea (the front of the eye). If untreated, this condition leads to the formation of irreversible corneal opacities and blindness [1].

Worldwide, there are about 8 million people irreversibly visually impaired by trachoma; an estimated 84 million cases of active disease in need of treatment, if blindness is to be prevented. It is open to all parties—governments, international organizations and non-governmental organizations—which are willing and ready to contribute to international efforts [2].

World Health Organization (WHO), in cooperation with various non-governmental organizations and national health services, recently began implementing a program to eliminate blinding trachoma—Global Elimination of Trachoma by 2020 (GET 2020) [3]. The program has adopted a strategy called SAFE, consisting of the following control measures: Surgery for entropion trichiasis, Antibiotics for infectious trachoma, Facial cleanliness to reduce transmission and Environmental improvements such as control of disease spreading flies and access to clean water [3].

The World Health Organization (WHO) recommends mass antibiotic treatment annually for at least 3 years of all individuals in any district or community where the prevalence of TF in children aged 1–9 years is at least 10 %. After three or more years of A, F and E interventions, the prevalence is reassessed and a decision is made regarding the need to continue or cease treatment [4].

Trachoma was endemic in 24 districts of Uganda in which the burden was higher in the semi-nomadic areas and this was associated with lower level of environmental hygiene. Prevalence of TF in children aged 1–9 years was 10.4 % in Bugin, 14.9 % in Mayuge, 57.1 % in Morroto and 57.8 % in Nakapiripirit.The proportion of the population with access to water source was 76.8 % in Bugin, 81.9 % in Mayuge, 44.8 % in Morroto and 43 % Nakapiripirit and the other environmental measure showed similar pattern [5].

The prevalence of trachoma in children <5 years prior to any mass treatment in Tanzania communities was 51 % trachomatous inflammation—Follicular (TF) and 1.6 % was trachomatous inflammation—Intense (TI). Reported coverage of the communities with annual mass treatment varied widely [6]. On the other hand a research done among 2777 children aged 1–9 years in two Gambia region showed that the prevalence of active trachoma was 12.3 % [7].

A population based cross-sectional surveys conducted in nine sites in Southern Sudan showed that prevalence of signs of active trachoma in children aged 1–9 years was: TF = 53.7 % TI = 42.7 % TF and/or TI = 64.1 % [8].

A research done in Gambia and Tanzania showed that, risk factors for follicular trachoma (TF) in both countries were ocular or nasal discharge, a low level of household head education, and being aged ≥1 year. Additional risk factors in Tanzania were flies on the child’s face, being amplicor positive, and crowding (the number of children per household) [9].

A nationwide survey conducted in Ethiopia in 2007 showed that the national prevalence of active trachoma (either TF or TI) for children 1–9 years of age was 40.1 %.The highest prevalence was registered in the regional states of Amhara (62.6 %), Oromia (41.3 %), and Southern Nations Nationalities Peoples (33.2 %). In major towns of the country, the prevalence of active trachoma is very low. The prevalence of active trachoma was also observed to be low in the Benshangul Gumuz and Afar regional states [10].

The 2012 Implementation guideline of The Carter Center showed that trachoma was common in Amhara region and among which West Gojjam had high burden of the cases with unknown factors and the prevalence of TT in adults was 10 % [11]. The high burden of trachoma in Amhara region calls for collecting a further district specific data and understanding of risk factors which is essential in designing appropriate interventions for the ‘A’, ‘F’ and ‘E’ components of the SAFE strategy. Hence, this study will reveal the current status of active trachoma in Gonji kolella district and estimate the need for intervention activities to eliminate active trachoma.

## Methods

### Study setting and design

**Fig. 1:**
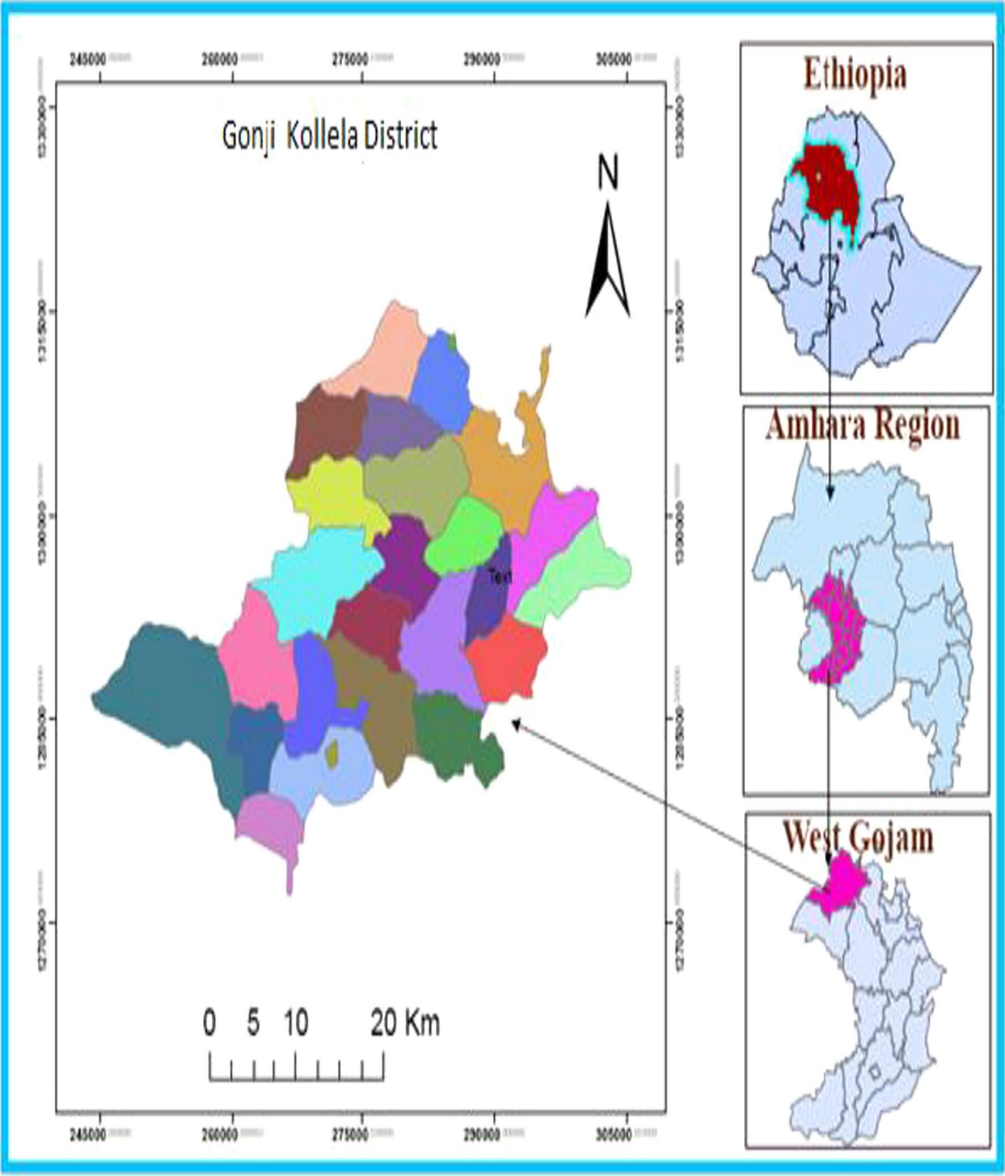
Map of the study area. (*Source* Land Administration office of Gonji Kollela district)

A community—based cross sectional study was used to determine the prevalence of active trachoma and associated factors among children aged 1–9 years, from December 2013 to June 2014. The study was conducted in Gonji kolella district, which is located 72 km far away from Bahir Dar, the capital town of Amhara region, to the Southwest direction. The total population of the district was estimated to be 119,482 of which 50.3 % are males and 49.7 % are female*s (*according to 2014 population estimation of the district) [12]. Of the total population 3305 were children 1–9 years of age. As to the health service facilities, there are 6 health centers, 26 health posts, several private and limited number of non-governmental health institution. The study subjects for this study were children whose age was between 1 and 9 years old found in the selected Keble’s of the district. This study was conducted in children aged 1–9 years because active trachoma is very common in small children than any other segment of the population [13]. Children who were unable to undergo physical examination for trachoma evaluation due to serious sickness during the study period were excluded from the study (Fig. 1).

### Sampling size and sampling procedure

Sample size was determined by single population proportion formula using EPI INFO stat calc program with the assumption of population size 119,482, 95 % level of confidence, 5 % of marginal error, and taking prevalence of active trachoma 24.1 % obtained from a previous community based survey [14]. Considering the design effect of 2, as the household in the rural areas are more or less similar in many characteristics such as household income, hygienic condition, access to safe and adequate water, and knowledge of disease associated with unhygienic conditions that showed no clustering in the study area and 10 % non-response rate, the final sample size became 618.

### Sampling procedure

Systematic sampling technique was used to select study participants. To get the individual sample units (subjects) at household level, a map of each Keble was taken from the Gonji Kollela Administration Office. The center of each Keble was located and to start the first house a lottery method was used. Once the direction was located using the lottery method, then it was started from the first household, which was immediately found from that direction and systematically continued by jumping every thirteen house unit to get next sample from the household. In households with more than one children, one among them was selected by lottery method.

### Data collection instrument and procedures

Data was collected using a pre tested structured questionnaire, observation (eye examination by using binocular loop) and self-declared for use of water or hygiene practice. Fifteen integrated eye care worker (IECW) clinical nurses who have been well trained by The Carter Center- Ethiopia on management and the way how to screen trachoma cases was assigned to perform eye examination by using two binocular examination loupes (×2.5). All of them are working in government health institutions in the district health center. Some of them had been participated on rapid assessment of trachoma, conducted in the region by The Carter Center- Ethiopia and they had taken more than 1 month training on eye examination.

Before starting the field work, data collectors and supervisors were trained for 2 days by the ophthalmologist (currently working in the regional referral hospital with experience of trachoma grading in the community) on the use of the WHO simplified grading system, sketch mapping and segmentation and overall data collection procedure. After the training, members were split into three teams.

### Trachoma grading

Each members was required to grade a standardized set of slides to identify clinical signs of trachoma. Only team members achieving more than 85 % overall agreement with the trainer’s gold standard diagnosis were selected to participate in the field examinations on the final day of training. The presence or absence of each sign was assessed separately for each eye of 50 children aged 1–9 years, who are not eligible, before the initiation of the main study. After reviewing the individual results of the team members, the trainer spent additional time with examiners on the final day during practical household survey training to confirm diagnoses. In order for a survey worker to be considered an examiner, she/he must have achieved an overall inter observer agreement of at least 85 % compared to the trainer during the field examinations. Only the best 2 members of the survey team were used as examiners.

#### Eye examination

**Fig. 2:**
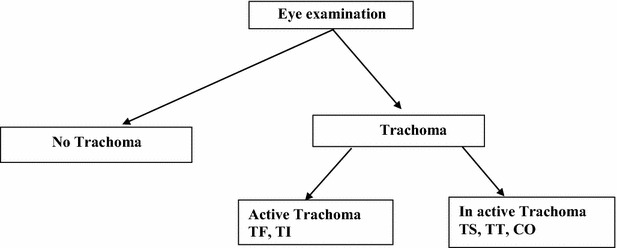
Schematic presentation of eye examination and result reporting procedure

All eligible children aged 1–9 years examined for active trachoma by IECW nurses especially trained for eye care. The steps of eye examination and trachoma grading strictly followed the WHO’s simplified grading system [6]. Both eyes were examined in the same sequence using a magnification convergent 2.5× binocular loupe adjustable to pupillary distance of the observer and a torch with spare batteries and a spare bulb were also used. Hygienic measures were also taken (the examiner was cleaning his/her hands with a spray bottle containing alcohol to clean between each examination) and results of the examination were registered (Fig. 2).

### Data quality control

To ensure the quality of the data, the questionnaire was pre-tested 1 week before the actual data collection time on 50 children aged 1–9 years in adjacent Keble’s^1^, which were not included in the study. During this time some questions were not understandable by the respondents. Therefore, necessary corrections were made and the questions were simplified based on the pretest findings. The questionnaire was prepared first in English and then translated into Amharic (the official language fluently spoken by all participants) for field work purpose and back to English for validation. Training was given for data collectors and supervisors. During the course of the data collection, facilitators were supervised at each site. The principal investigator was also closely supervising the field activity on daily basis and was checking the completeness of filled questionnaire and whether recorded information makes sense to ensure the quality of data collected. Questionnaire templates were prepared and customized to avoid entry of illegal values and skip patterns.

### Data processing and analysis

Data clean up and cross-checking was done before analysis. Data were checked, coded and entered to EPI INFO version 7 then it was exported to SPSS version 20 for analysis. Both descriptive and analytical statistical procedures were utilized. Descriptive statistics like percentage, mean and standard deviation were used for the presentation of demographic data and prevalence of active trachoma. Binary logistic regression was used to identify factors associated with active trachoma among children aged 1–9 years. Multiple logistic regression model was used to identify the proximate determinants of the known risk factors by controlling the possible confounding effects of other variables and finally the variables which had independent association with active trachoma were identified on the basis of Odds Ratio, with 95 % CI and *p* value less than 0.05. The variables were entered to the multivariate model using the backward stepwise regression method. The necessary assumption of the logistic regression model was checked by Hosmer and Lemeshow goodness of fit test statistics (p value = 0.77).

### Ethical approval

Ethical clearance was obtained from Ethical review committee of GAMBY College of medical science. Permission to conduct the research was obtained from the district. Written Informed consent was obtained from care takers of the children who were selected to participate in the study after explaining the purpose of the study. Finally, children who were found to have active trachoma (diagnosed as TF, TI) were provided with the standard treatment free of charge and complicated cases were referred to the nearest health center for better treatment.

## Results

### Socio demographic characteristics of the respondents

A total of 618 children aged 1–9 years participated in the study, giving response rate of 100 %. Most 353 (57.1 %) were male. Mean age of the sample population was 4.96 [standard deviation (SD) ± 2.42] years. The age distribution showed that, out of the total study subjects, 364 (58 %) were within the age group between 1 and 5 years and children between 6 and 9 years of age comprised 41.1 %.

More than half of the study subjects (57.1 %) live in households headed by male. Great majority (95 %) of heads of households were farmers by occupation. In the study area, the average family size among the study participants was from 4 to 6 and the mean family size was 5.31 (SD ± 1.288) persons. More than half of the respondents (51.1 %) live in households with one to two room. Distribution of the study sample by literacy status among the heads of households showed that, 41.3 % were illiterate and 58.7 % were able to read and write. In this study, 342 (55.3 %) households had less or equal to two children age less than 10 years. From the total study participants, only 29.8 % children were enrolled at school for the academic year (Table 1).

**Table 1:** Socio-demographic characteristics of study participants in rural communities of Gonji kollela District, West Gojjam Zone, ANRS, Northwest Ethiopia, 2014

Characteristic	Number (618)	Percentage (%)
Sex of HH head		
Male	353	57.1
Female	265	42.9
Educational Status of HH head		
Can read and write	363	58.7
Can’t read and write	255	41.3
Occupation of HH head		
Farmer	587	95
Other	31	5
Number of family member		
≤5	433	70.1
>5	185	29.9
Number of <10 year children family member		
≤2	342	55.3
≥3	276	44.7
Number of living room		
1–2	316	51.1
≥3	302	48.9
Age of selected child		
1–5	364	58.9
6–9	254	41.1
Sex of selected child		
Male	353	57.1
Female	265	42.9
Educational status of the child		
Preschool	413	66.8
Attending	184	29.8
Can’t read and write	21	3.4

### Environmental and behavioral factors of study participants

Family members of 433 (70.1 %) children cook their food in the separate room from where the family live while those families of 185 (29.9 %) children cook in the field. Those children who live in households with cooking rooms having windows constitute 255 (58.9 %). The common sources of water for domestic consumption in the study area were pipe water for 434 (70.2 %) and protected well for 115 (18.6 %) of the study children. Only 30 (4.9 %) get their water from unprotected spring. Families of 516 (83.5 %) children in the study areas get their water traveling for less than 30 min. For families of 102 children (16.5 %), the water source was from 30 min to two hour away from their home. In this area, the larger proportion of children 397 (64.2 %) live in households where the average daily water consumption is 60–80 l/family. In the study Keble’s, families mostly dispose their domestic wastes by burning and burying 439 (71 %) and the rest 29 % dispose openly. In the study area, families of 590 (95.5 %) children have pit latrine. Among study participant, families of 352 (59.6 %) children were utilizing latrine. Out of the total children, great majority, 525 (85 %) were members of the families who have cattle and 181 (34.5 %) of them live in same rooms where the cattle’s are kept. Face washing practice of the study participant were assessed by direct observation and self-response. More than half of the study children 368 (59.5 %) wash their faces twice and above per day, while 250 (40.5 %) wash their face once per day. Majority of study children do not use soap for face washing. Only 248 (40.1 %) of children use soap (Table 2).

**Table 2:** Environmental and Behavioral factors of study participants in rural communities of Gonji kollela District, Northwest Ethiopia, 2014

Characteristic	Number (618)	Percentage (%)
Source of water		
Pipe	434	70.2
Protected well	115	18.6
Protected spring	39	6.3
Unprotected spring	30	4.9
Time to obtain water		
≤30 min	516	83.5
30 min–2 h	102	16.5
Amount of water used per day		
60–80 l	397	64.2
20–40 l	187	30.3
Greater than 80 l	25	4
Less than 20 l	9	1.5
Place where cook food		
Separately in the living room	433	70.1
In the field	185	29.9
Presence of window in the cooking room (n = 433)		
No	178	41.1
Yes	255	58.9
Mechanism of disposing dry waste		
Proper	439	71
Improper	179	29
Presence of latrine		
Yes	590	95.5
No	28	4.5
Utilization of latrine		
Yes	352	59.6
No	238	40.3
Owner ship of cattle		
Yes	525	85
No	93	15
Place where cattle passing night (n = 525)		
In the living room but separately	344	65.5
Cattle house	181	34.5
Frequency of washing face (selected child)		
Two times and above per day	368	59.5
Once per day	250	40.5
Using of soap when wash face		
No	370	59.9
Yes	248	40.1

### Prevalence of active trachoma

Enumerated children aged 1–9 years were examined for signs of trachoma. The signs of active trachoma, TF and TI, were observed in children 1–9 years of age regardless of gender. Overall prevalence of active trachoma among children aged 1–9 years in rural communities of Gonji Kollela district was found to be 23.1 % (Trachomatous follicles—TF, in 22.5 % (95 % CI : 22.3–22.69 %); Trachomatous inflammation—TI, in 0.6 % (95 % CI: 0.4–0.79 %).

Of the total 364 children aged between 1 and 5 years, 87 (23.9 %) had active trachoma while the figure of age group between 6 and 9 years was 254 children, of which 56 (22.04 %) had active trachoma.

### Factors associated with active trachoma

Bivariate and multivariate logistic regression analysis of possible explanatory variable of TF and TI was carried out on socio-demographic and other characteristic. Variable that fulfill the requirement (p < 0.2) in the bivariate analysis were analyzed for multivariate logistic regression.

Multivariate logistic regression analysis constructed by including the factors found to be significant in the bivariate analysis, showed that, Family size (>5) (AOR = 14.32, 95 % CI 6.108–33.601), number of children under 10 years of age within household (AOR = 25.53, 95 % CI 9.774–66.686), latrine utilizations (AOR = 10.274, 95 % CI 4.274–24.968), route of waste disposal (AOR = 3.717, 95 % CI 1.538–8.981), household literacy (AOR = 2.892, 95 % CI 1.447–5.780), cattle housing practice (AOR = 4.75, 95 % CI 1.815–12.431), time to collect water (AOR = 25.530, 95 % CI 8.995–72.461), frequency of face washing practice (AOR = 6.384, 95 % CI 2.860–14.251) and source of water (AOR = 2.353, 95 % CI 1.134–4.882) were significant predictors of presence of active trachoma among respondents (Table 3).

**Table 3:** Bivariate and multivariate logistic regression analysis of factors associated with active trachoma among children aged 1–9 years, Gonji Kollela District, Northwest Ethiopia, April 2014

Characteristics	Presence of active trachoma		OR with 95 % CI	
	Yes	No	Crude	Adjusted
Number of family members				
≤5	68	365	1.00	1.00
>5	75	110	3.660 (2.475–5.413)**	14.326 (6.108–33.601)**
All family members use latrine				
No	81	185	2.048 (1.402–2.991)**	10.274 (4.277–24.968)**
Yes	62	290	1.00	1.00
Educational status of house hold head				
Can’t read and write	84	171	1.942 (1.331–2.834)*	2.892 (1.447–5.780)*
Can read and write	59	304	1.00	1.00
Number of less than ten year				
1–2	41	301	1.00	1.00
≥3	102	174	4.304 (2.863–6.470)**	25.53 (9.774–66.686)**
Frequency of face washing				
≥2 times per day	71	297	1.00	1.00
Once per day	72	178	1.692 (1.161–2.466)*	6.384 (2.860–14.251)**
Time to collect water				
≤30 min	68	448	1.00	1.00
>30 min	75	27	18.30 (11.006–30.430)**	25.530 (8.995–72.461)**
Waste disposal				
Proper	78	361	1.00	1.00
Improper	65	114	2.639 (1.785–3.901)**	3.717 (1.538–8.981)*
Cattle passing night				
In the living room but separately	123	221	9.517 (4.847–18.687)**	4.75 (1.815–12.431)*
Cattle house	10	171	1.00	1.00
Source of water				
Pipe	65	369	1.00	1.00
Other	78	106	4.177 (2.818–6.193)**	2.353 (1.134–4.882)*

(n = 618)

*COR* crude odds ratio, *AOR* adjusted odds ratio

* Statistically significant at P < 0.05

** Statistically significant at P < 0.001

## Discussion

The major objective of this study was to assess the current status of active trachoma, and to identify associated factors among children aged 1–9 years in rural communities of Gonji Kollela. In this study, the prevalence of active trachoma was found to be 23.1 % of which 22.5 % TF and 0.6 % TI. It varied from 23.9 % in children aged between 1 and 5 years to 22 % in 6–9 years. This finding was lower than previous studies conducted in Amhara region, 62.6 % [10]. It was also lower when compared with studies in other regions of Ethiopia [13–17]. The difference could be attributed to the expanding of health service coverage and utilization of health development armies to heighten awareness of caretakers on active trachoma.

According to the WHO recommendation, the result of current study indicates that active trachoma was still a public health problem in rural communities of Gonji Kollela district. The prevalence of active trachoma in children aged 1–5 years was 23.9 % and it started to decline to 22 % in the age group between 6 and 9 years. The finding is consistent with other studies in Guragie zone [18], South Wollo zone [19] and Sidamo region [20]. Since, children aged 1–5 years are the main infectious pool (reservoir for disease). This could be the possible causes for the higher prevalence of active trachoma among children of 19 years.

Risk factors for the development of trachoma were also compatible with other studies in other parts of the country. The current study shows that children living in households with family size of greater than or equal to five are 14 times more likely to have active trachoma than children living in households with family size less than five (p < 0.001, AOR = 14.326, 95 % CI = 6.108–33.601).

In the current study, there is significant association between utilizing latrine and the occurrence of active trachoma. Families that didn’t use latrine were 10.2 times more likely to be affected by active trachoma than those families who utilize latrine (p < 0.001, AOR = 10.274, 95 % CI = 4.277–24.968). The finding is in line with study done by WHO [21]. Even though the association of the presence of a functional latrine near the house with lower trachoma prevalence has been detected by many investigators, the mechanism by which it would decrease trachoma is not entirely clear. Some say that the presence of latrine may reduce eye-seeking flies in the surrounding environment.

The study also found that active trachoma was significantly associated with face washing practice. Children who wash their face once per day were 6.4 times more likely to be affected by active trachoma as compared to those who wash their face greater than or equal to 2 times per day (p < 0.001, AOR = 6.384, 95 % CI = 2.860–14.251). A study among children in Uganda [5] and Wereilu district [22] also came up with the same finding that, high prevalence of active trachoma were observed in children with poor face washing habit and the burden is higher in the semi-nomadic areas.

The result of this study indicated that, educational status of household head had significant association with active trachoma of children. Children from households whose family heads can’t read and write were 2.89 times more likely to be affected by active trachoma than children from household whose family heads can read and write, the difference was statistically significant. This finding is supported by other studies conducted in Gambia and Tanzania [23] which found that the influence of educational status of heads on children face washing habit or because literacy status is directly related to important socio-demographic factors such as family size.

Active trachoma is more prevalent in rural population with low socio-economic status, without good water supplies and basic sanitation services than urban population with relatively better economy and better accessibility to water supply and sanitation [20, 24, 25]. In this study more active trachoma cases were identified in families who have children greater than or equal to three <10 years, family size >5, family members who didn’t use latrine facility properly and distance of primary source of water with greater than half an hour (AOR = 25.530, 14.326, 10.274, 25.53 respectively, p < 0.001). The findings of this study revealed that, the risk for infection did vary with the distance to collect water for domestic consumption or source of water for domestic consumption and mechanism of waste disposal. This could be due to proper waste disposal and water source are important in the study area, when compared to other factors for the transmission of active trachoma.

The prevalence of active trachoma was lower in households disposing their domestic wastes by burning than in those disposing in other way (in the open field) and the difference was statistically significant (AOR = 3.717, p = 0.004). The finding agrees with the study conducted in Sidamo [18]. The case of active trachoma was mainly high in families who have had the habit of co-habitance of people with domestic animals (AOR = 4.75, CI = 1.815–12.431). This finding agrees with the research done in India [26].

In this particular study; cattle ownership was not determinant factor for the occurrence of active trachoma. This agrees with the study conducted in Southern Ethiopia [18].Trachoma was more common in families who pass nights with their cattle in the same room. But keeping other potential risk factors constant, the association of cattle housing practice with active trachoma was statistically significant.

### Limitation of the study

This study has some important limitations that should be kept in mind when interpreting the results. First, the cross-sectional nature of the study design does not confirm definitive cause and effect relationship. Furthermore, the study may prone to reporting bias since some of the data was collected based on self-reported information. Finally; this study did not use the qualitative method.

## Conclusion

The prevalence of active trachoma among rural communities of children aged 1–9 years was found to be high in reference to WHO recommended thresholds to initiate trachoma control recommendation (>10 % prevalence), which indicates that active trachoma is still a major public health concern in the study area. Therefore, it is recommended that coordinated work on implementing the WHO endorsed SAFE strategy in particular and enhancing the overall living conditions of the community is crucial.

## Abbreviations

TF: trachomatous inflammation-follicular
TI: trachomatous inflammation-intense
TS: trachomatous conjunctival scarring
tt: trachomatous trichiasis
CO: corneal opacity

## Footnotes

1 Keble is the smallest administrative unit of the Federal Democratic Republic of Ethiopia.
